# Motor Neuron Disease in a Dish: The Power of Human Induced Pluripotent Stem Cell-Derived Spinal Motor Neurons and Glial Cells

**DOI:** 10.3390/cells15191798

**Published:** 2026-10-01

**Authors:** Martina Morrone, Chiara F. Valori, Liliana Brambilla, Daniela Rossi

**Affiliations:** Istituti Clinici Scientifici Maugeri IRCCS, Laboratory for Research on Neurodegenerative Disorders, 27100 Pavia, Italy; martina.morrone@icsmaugeri.it (M.M.); chiaraflora.valori@icsmaugeri.it (C.F.V.); liliana.brambilla@icsmaugeri.it (L.B.)

**Keywords:** induced pluripotent stem cells, Amyotrophic Lateral Sclerosis, motor neurons, astrocytes, *in vitro* models

## Abstract

Induced pluripotent stem cells (iPSCs) have revolutionized disease modelling by providing unprecedented opportunities to investigate complex human disorders. Among others, Amyotrophic Lateral Sclerosis (ALS), the most common adult-onset motor neuron disease, clearly embodies this complexity. This disorder remains particularly challenging not only because of its marked clinical and molecular heterogeneity but also as a consequence of the composite interactions between motor neurons and the neighboring non-neuronal cells, especially astrocytes, which contribute to disease progression. The ability to generate patient-specific spinal motor neurons and astrocytes from human iPSCs has enabled the development of *in vitro* models that recapitulate key pathological features of ALS, offering valuable platforms to investigate disease mechanisms, to identify therapeutic targets, and to support personalized medicine approaches. In this review, we summarize current iPSC-based strategies for modelling ALS, highlighting their applications in studying disease physiopathology and drug development. In addition, advanced human iPSC-derived technologies, including organoids and organ-on-a-chip systems, are discussed highlighting their potential, current limitations, and future perspectives.

## 1. iPSCs: Establishing a Molecular Bridge Between Pluripotency and Differentiation

Chronic, late onset neurodegenerative diseases are personal tragedies to patients and their families. Moreover, due to the growing aging of the world population, they represent an increasing burden to the health systems of Western Countries. Although treatment options are still limited and unable to halt neurodegeneration, the development of human-centered models, during the last 20 years, has provided unprecedented opportunities for drug discovery. Here, we discuss how the generation of induced pluripotent stem cells (iPSCs) has unlocked novel possibilities to gain insights into disease pathogenesis and to test therapeutic options. In spite of several hurdles that still need to be overcome, we debate how iPSC-based models are revolutionizing research on neurodegenerative disorders, with a special focus on Amyotrophic Lateral Sclerosis (ALS), the most common motor neuron disease.

### 1.1. The Discovery of iPSCs Revolutionized Biomedical Research

Accurate modelling of complex diseases is a prerequisite for developing effective therapeutic agents and represents, at the same time, a challenge that needs constant refinement. In this regard, human stem cells (SCs) attracted great attention because they harbor the genetic make-up of the desired patient group and have the ability to self-renew or, when properly guided, can give rise to cells of different embryonic lineages. Overall, these features make them ideal candidates for both disease modelling and cell therapy implementation (for an in-depth discussion of SC classification and differentiation potential, see Appendix A). However, the widespread use of human embryonic stem cells (ESCs), the first known source of human SCs, was hampered by ethical implications related to their generation, which entails destroying human embryos, as well as by the risk of inducing immune rejection when used for transplants [1].

Importantly, research on SCs significantly flourished with the discovery of iPSCs in 2006, which allowed circumventing the ethical issues associated with ESCs and granted the possibility of modelling late-onset, sporadic disorders [2]. These key attributes arise from the nature of iPSCs which retain the pluripotency feature of natural ESCs, albeit originating from differentiated adult somatic cells. This breakthrough revolutionized the concept of irreversible cellular differentiation, a discovery that led Prof. Shinya Yamanaka to be awarded the Nobel Prize for Medicine and Physiology along with Sir John B. Gurdon (https://www.nobelprize.org/prizes/medicine/2012/summary/ accessed on 28 September 2026). 

Briefly, building on knowledge gained from studies on ESCs, Takahashi and Yamanaka identified a minimal combination of four transcription factors, i.e., octamer-binding transcription factor 4 (Oct4), Sex-Determining Region Y-Box transcription factor 2 (Sox2), Krüppel-like factor 4 (Klf4), and c-Myc—later referred to as the “Yamanaka factors” or “OSKM factors”—whose ectopic expression was found to induce the reprogramming of murine and human fibroblasts toward a pluripotent-like state [2,3]. It soon became evident that iPSCs held the greatest potential as tools for biomedical research, thus prompting the need to implement novel methods to induce pluripotency, as discussed in Appendix B. 

### 1.2. Opportunities and Advantages

One of the most revolutionary aspects of iPSC technology consists of the possibility of generating human cell models, capable of reproducing pathological characteristics directly associated with the patient genetic profile, a particularly relevant feature for disorders caused by complex genetic rearrangements or with a polygenic basis. Therefore, iPSCs allow addressing a specific disease in the genetic context in which it develops naturally, thus complementing traditional animal models, which can still be useful for studying complex systemic responses, nervous system interactions and some other aspects of the human physiopathology [4,5]. Moreover, another benefit of these cell models is that they allow the analysis of early molecular and cellular alterations preceding the onset of clinical symptoms. In the context of neurodegenerative diseases, this is a crucial aspect, particularly if we compare it with data arising from the analyses of post-mortem tissues. The latter indeed allow investigating only processes related to the terminal stage of the disease, when many pathways and cellular structures are largely deranged [5]. However, a prerequisite for exploiting this potential advantage consists of the ability to differentiate iPSCs into the desired cell type. Considerable efforts have been devoted to developing protocols apt to obtain the cell population of interest. For the Central Nervous System (CNS), strategies have been optimized to guide iPSCs toward the specific neuronal subtypes (i.e., cholinergic, glutamatergic, etc.) and/or glial populations that are mostly affected in the disease of interest (reviewed in [6,7,8,9,10,11]).

In addition to their usefulness as tools for disease modelling, iPSCs opened new perspectives in the fields of regenerative medicine and cell therapy. Several clinical trials, involving cellular derivatives of iPSCs, have been undertaken in the context of various diseases characterized by the irreversible loss of specific cell populations [12]. Remarkably, in March 2026, the Ministry of Health, Labour and Welfare of Japan granted conditional marketing authorization to two therapies based on iPSC-derived cells, i.e., cardiomyocytes for heart failure and dopaminergic neurons for Parkinson’s disease (PD), in spite of concerns about the clinical data supporting the decision [13].

Another opportunity that is offered by iPSCs is experimental flexibility. While differentiation toward a specific cell type is a suitable strategy to investigate cell-autonomous events, combining different cell populations into co-cultures permits to studying their interplay. However, interrogating cytological and network intricacies of tissues requires more complex 3-dimensional (3D) models. This need is particularly compelling to model the CNS, where cellular diversity, connectivity and regional organization all contribute to its extraordinary function. To this end, Lancaster et al. [14] discovered that iPSCs can self-organize into 3D structures recapitulating a complex multicellular architecture, with different cell types arranged in a spatial organization that resembles the *in vivo* brain structure. These cultures were named cerebral organoids and they were obtained in the absence of any patterning signal, simply under the necessary culturing conditions for intrinsic iPSC cues to influence development [14]. Since that groundbreaking finding, the field has constantly evolved with new protocols, including those exploiting guiding molecules to obtain region-specific organoids and connecting them into assembloids and connectoids (reviewed in [15]).

### 1.3. Challenges and Limitations

Despite their success, the clinical translation of iPSC-derived products and their ability to model complex, late-onset diseases revealed several challenges, the most severe being cellular heterogeneity. The latter is a broad concept that includes immaturity and low fidelity of differentiated cells when compared with their intended somatic counterparts. The occurrence of such events was confirmed by transcriptome analyses at the single-cell level (scRNAseq) of 2-dimensional (2D) [16] and 3D iPSC-derived models [17]. These studies not only revealed the coexistence of a continuum of cells with an increasing level of maturation in these preparations but also the presence of cells diverging from the intended differentiation path, thus urging the development of computational tools for proper identity assignment [18,19]. These findings have practical implications as they argue that the interpretation of bulk population studies should be attentively evaluated because the observed results recapitulate the contribution of different cell types. Furthermore, these features of iPSCs pose the risk of tumorigenicity and immunogenicity in clinical settings [1,20]. In particular, the fact that iPSCs are able to proliferate indefinitely and to generate many diverse cell types can represent a shortcoming after transplantation. More specifically, even if a few undifferentiated cells remain in the final cell products and are transplanted into patients, they may continue to divide uncontrollably, ultimately forming tumors [21,22]. To overcome this issue, methods for selection, identification and removal of undifferentiated cells have been implemented [23,24]. In addition, significant effort has been made to develop methods to generate iPSCs safely and efficiently (see Appendix B). Procedures that implicate transient expression of reprogramming factors were deemed safer than techniques based on the use of integrating vectors, as the untimely reactivation of the latter may promote tumor formation. The risk of developing tumors is also associated with genetic alterations that may occur during cell expansion in culture. This highlights the need for developing more efficient methods to increase the differentiation efficiency and, as a consequence, the purity of the final cell product.

Although, patient-derived iPSCs were initially expected not to be subject to immune rejection, studies have shown that immune responses may occur. Indeed, the Altered Genome Induced Immune Response (AGIIR) hypothesis [25] has proposed that genetic and epigenetic changes, occurring during cell reprogramming or differentiation, can potentially affect how transplanted cells are recognized by the immune system [1]. In this context, studies have demonstrated that a high iPSC passage number may introduce genetic alterations, resulting in reduced differentiation efficiency [26]. Lastly, different iPSC lines may show considerable variability depending on the donor, the reprogramming method, and the differentiation protocol used. As a result, cells generated from different lines may not behave in the same way, making it difficult to compare results between studies and to establish standardized protocols [6,27]. Attempts have been made to minimize the impact of variability and to obtain results that truly reflect disease-associated phenotypes. These include increasing the number of differentiation batches to dampen procedure-associated reproducibility issues [16]. An alternative, intriguing strategy consists of pooling cells from different individuals to generate cultures known as iPSC villages and differentiating the cells in 2D or even 3D models. While diminishing batch effects, this approach requires intensive computational analysis to deconvolve donor-specific contributions as well as to monitor proliferation rates, differentiation efficiencies and intercellular crosstalk within each culture dish [28,29,30,31,32]. Complementarily, the development of the Clustered Regularly Interspaced Short Palindromic Repeats (CRISPR)/CRISPR-associated protein (Cas) technology enabled scientists to manipulate the genome of target cells with unprecedented flexibility (reviewed in [33]). When applied to iPSCs, this tool allowed the generation of isogenic lines, in which pathogenic mutations can be either corrected in patient cells to restore the native sequence or introduced in healthy lines to isolate the effect of the genetic variant from inter-donor variability [34]. Of note, more advanced genome editing strategies hold the potential for even greater precision and versatility, although their application to manipulate iPSCs is still in its infancy [35].

Additional limitations of iPSCs include (1) the high costs of production, maintenance and differentiation, which might not be completely covered by the budget constraints of funding bodies, leading to the inevitable reduction in the number of lines included in individual studies, and (2) the differentiation protocols, which are typically labor-intensive and time-consuming. Moreover, it was previously demonstrated that the transcriptomic profile of human iPSC-derived terminally differentiated neurons is closer to that of fetal (rather than adult) primary brain cells. More specifically, these analyses pointed out that iPSC-derived cortical neurons exhibit undetectable levels of many neuronal adult markers, thereby emphasizing their immature nature compared with primary brain neurons [36,37]. Similar observations were also reported for spinal motor neurons [16]. One possibility to explain these features is that cellular characteristics associated with aging may be lost during reprogramming, making it more challenging to reproduce disease phenotypes that develop over decades in patients [6,38]. In particular, the establishment and maintenance of pluripotency are strictly associated with the reversal of the epigenetic barriers acquired during development. At the molecular level, these modifications consist of DNA demethylation processes and histone modifications, which alter chromatin architecture and lead to deep re-configuration of the cellular transcriptional networks associated with pluripotency [27]. Different approaches are currently being investigated to force aging in iPSC-derived models, including genetic manipulations of key transcripts. For example, Miller and colleagues found that the expression in iPSC-derived fibroblasts and neurons of progerin, a truncated form of lamin A associated with premature aging, was able to induce multiple aging-related markers and characteristics [39]. Alternatively, iPSCs can be manipulated to harbor shortened telomeres and then differentiated to generate spinal motor neurons and astrocytes, as well as spinal cord organoids. Cells in these models displayed signs of aging, including reduced soma size, reduced neurite complexity and enhanced DNA damage [40]. Telomere shortening can also be achieved with the administration of a pharmacological agent, an approach that had been successfully applied to generate aged dopaminergic neurons as models of PD [41]. Because other chemicals have been tested to refine models of other neurodegenerative disorders, we envision that similar investigations may soon be extended to ALS cells [42,43,44]. Finally, the pioneering field of space medicine revealed that microgravity holds the potential to accelerate aging in iPSC-derived cells, thereby preventing any potential confounding effects of genetic manipulations or chemical administration (reviewed in [45]). In the context of CNS research, Marotta et al. compared the transcriptional profile of neural organoids derived from a patient with primary progressive multiple sclerosis and a patient with PD with the respective control cases, either grown on Earth or on the International Space Station for 30 days. Intriguingly, organoids grown in the lower Earth orbit displayed a more advanced status of maturation, showing upregulation of transcripts associated with mature neurons and glial cells as well as downregulation of proliferation and stemness markers, respectively [46].

Exposure to pollutants and environmental conditions is another key factor that can act in synergy with aging to contribute to late-onset disorders, such as neurodegenerative conditions [47,48]. Interestingly, iPSC-derived 2D and 3D models provided insightful evidence from a neurotoxicology standpoint (reviewed in [49]). Yet, the interaction between the environment, genetics and aging is a timely topic that has not yet been investigated, a gap most likely due to the extreme complexity of the experimental settings required to answer this question. 

Altogether, this amount of evidence suggests that continuous improvements in differentiation protocols, quality control, and cell maturation strategies are essential to fully attain the human disease modelling and iPSC therapeutic potential. Additional work is necessary, especially for tackling complex diseases, such as neurodegenerative conditions, which are currently incurable.

**Figure 1:**
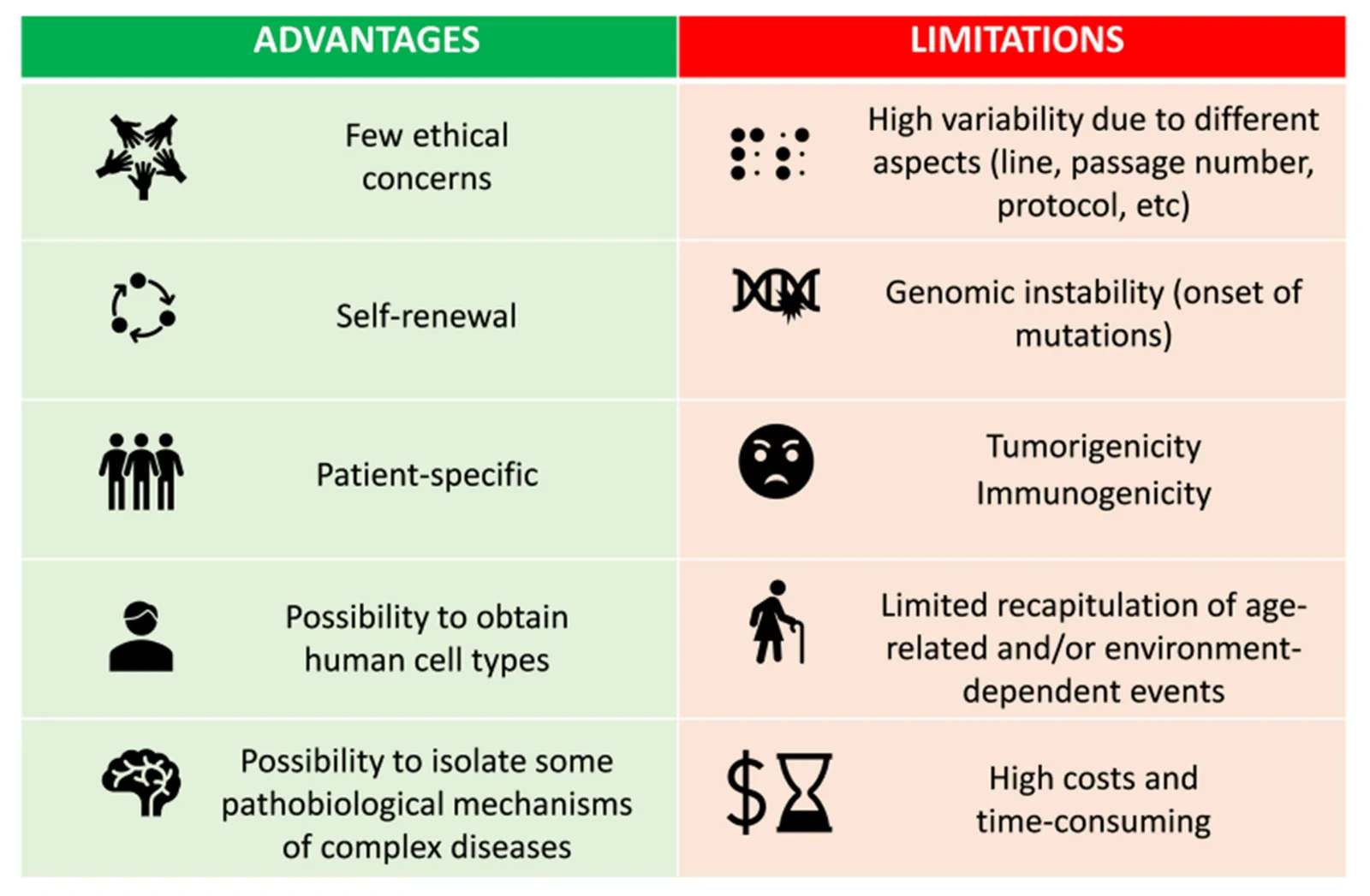
Advantages and limitations of iPSC technology. Key advantages of iPSCs include reduced ethical issues compared to ESCs, an unlimited source of cells due to their self-renewal ability, patient specificity, and the ability to generate diverse human cell types for disease modelling. Major limitations include issues of reproducibility associated with the number of iPSC passages, line to line variability, genomic instability, tumorigenic and immunogenic potential, challenges in modelling age- and/or environment-related features, high costs, and lengthy protocols required for iPSC culture maintenance and differentiation. Image created by Microsoft Power Point 2019.

General advantages and limitations of iPSCs are summarized in Figure 1. 

## 2. Motor Neuron Diseases: From Neuronal Degeneration to Network Dysfunction 

Among neurological disorders, motor neuron diseases (MNDs) represent an area in which iPSC technology has been extensively exploited to investigate the mechanisms underlying the diseases. MNDs are a group of severe neurological disorders characterized by the degeneration of upper motor neurons (UMNs), localized in the motor cortex of the brain, and/or lower motor neurons (LMNs), confined to the brainstem and in the ventral horns of the spinal cord. Degeneration of UMNs results in muscle stiffness, spasticity, hyperreflexia and increased muscle tone. Voluntary movements become slow and poorly coordinated, while muscle strength may initially be relatively preserved. Primary lateral sclerosis (PLS) represents a disorder characterized by UMN degeneration. In contrast, the degeneration of LMNs disrupts the final pathway responsible for muscle contraction, leading to progressive muscle weakness, fasciculations, hyporeflexia or areflexia, and atrophy. As denervation progresses, affected muscles lose mass and function, causing increasing disability. Progressive muscular atrophy (PMA) and spinal muscular atrophy (SMA) are examples of disorders primarily involving LMNs, while ALS combines the degeneration of both upper and lower motor neurons. Consequently, patients present a mix of UMN and LMN signs. Symptoms usually begin focally, affecting either the limbs or bulbar muscles, and progressively extend to other regions. As the disease advances, patients develop severe motor impairment, dysarthria, dysphagia, and respiratory insufficiency, which is the leading cause of death [50,51].

A global understanding of ALS epidemiology is challenging due to the rarity of the disease and its rapid progression. The worldwide incidence of ALS is estimated to be approximately 1–2 cases per 100,000 individuals per year, while prevalence ranges from 4 to 8 cases per 100,000 people, although geographical differences have been reported [52,53]. The disease typically develops between 40 and 70 years of age and shows a slightly higher incidence in males than females. Most patients survive between 2 and 5 years after symptom onset, although disease progression is highly variable and a small proportion of patients (5–10%) may survive for more than a decade [54].

The etiology of ALS is complex and involves genetic, epigenetic and environmental factors, which make it a multifactorial disease. Approximately 90–95% of cases are classified as sporadic ALS (sALS), with no clear family history, whereas the remaining 5–10% are familial forms (fALS), associated with inherited genetic mutations. Over the last three decades, significant advances in human genetics have identified several genes associated with ALS pathogenesis. Among them, mutations in the *Superoxide Dismutase 1* (*SOD1), Chromosome 9 Open Reading Frame 72 (C9orf72)*, *Transactive Response DNA-Binding Protein* (*TARDBP*, coding for TDP-43), and *Fused in Sarcoma* (*FUS*) genes account for 50-70% of familial cases and have provided important insights into disease mechanisms [55]. In particular, hexanucleotide repeat expansions in *C9orf72* represent the most common genetic cause of ALS in European populations, whereas mutations in the *SOD1* gene were the first genetic alterations linked to the disease to be identified and remain among the best-characterized forms of inherited ALS [56,57,58]. Although genetic factors are major players in the pathogenesis of ALS, their role is inevitably interconnected with epigenetic processes, such as DNA methylation/demethylation and histone modifications. In addition, a link exists with some environmental factors, including exposure to pesticides, heavy metals and high-intensity radar waves. All these factors, in a currently unknown combination, induce a wide array of aberrant intracellular and intercellular mechanisms ultimately contributing to ALS pathogenesis. These include protein misfolding and aggregation, impaired RNA metabolism, mitochondrial dysfunction, oxidative stress, axonal transport defects, altered protein homeostasis, excitotoxicity, neuroinflammation, and defects in neuron–glia communication [59].

Despite considerable advances in the knowledge of ALS biology, therapeutic options remain limited, making ALS one of the most aggressive and devastating neurodegenerative disorders. Currently, only a few disease-modifying treatments have been approved by regulatory bodies; these drugs are able to reduce glutamate-mediated excitotoxicity (Riluzole) [60], to fight oxidative stress (Edaravone) [61], and to reduce SOD1 protein levels and neurodegeneration biomarkers in patients carrying *SOD1* mutations through an antisense oligonucleotide (Tofersen) [62]. Nevertheless, these treatments provide only limited benefits, highlighting the urgent need for more effective therapeutic strategies and reinforcing the importance of disease-relevant human models for drug discovery and personalized medicine. A major challenge in the development of such approaches is the incomplete understanding of the precise molecular mechanisms underlying disease onset and progression, as well as the limited knowledge of the mutual regulation and interaction between the cellular populations affected by the disease.

## 3. Modelling ALS in a Dish: Differentiation of Human iPSCs into Motor Neurons 

The possibility of generating specific neural populations from patient-derived iPSCs has profoundly transformed research on neurodegenerative disorders, including ALS. These methods (reviewed in [7,8]), allowed to generating distinct cellular models that faithfully recapitulate key pathological features, enabling the investigation of early cell-autonomous pathogenic events of the disease. Up to now, animal models of ALS have provided critical insights about the molecular pathways underlying the disease; they have taught us to interpret complex genotype-phenotype correlations as well as served as systems to screen new potential therapeutics. These *in vivo* models were frequently generated through the transgenic overexpression of specific mutant genes implicated in ALS etiology, including *SOD1*, *TARDBP*, *FUS*, and valosin-containing protein (*VCP*) [63]. However, it is crucial to note that not only are small percentage of ALS cases associated with genetic mutations, but also different affected families can carry distinct mutations in the same gene, thereby introducing a further level of complexity. This represents a major intrinsic limitation of the mammalian *in vivo* models, which cannot be established for such a wide number of genetic variants. In addition, they fail to recapitulate sALS forms that constitute the vast majority of clinical cases. Thus, while murine models exhibit severe shortcomings in mimicking sALS, the versatility of iPSCs allows the investigation of both familial and sporadic forms of ALS and, for each of them, generating cellular models of the desired cell populations. In the next subparagraphs, we will discuss how iPSCs can be guided into motor neurons and how these models can move ALS research forward.

### 3.1. Modelling LMNs

In a seminal article, Dimos and colleagues [64] were the first to generate and describe spinal motor neurons starting from iPSCs derived from an 82-year-old woman diagnosed with a familial form of ALS. Through the use of two small molecules involved in the neurulation process, they obtained β-tubulin III-positive cells that co-expressed the homeobox HB9 gene (also known as MNX1 or Motor Neuron and Pancreas Homeobox 1), a key transcription factor important for spinal motor neuron specification and maintenance. More specifically, they used retinoic acid (RA), which promotes caudalization to acquire spinal cord specification, and an agonist of the sonic hedgehog (SHH) signaling pathway, which drives ventralization, to restrict neural progenitor identity toward the motor neuron lineage [65]. After this study, additional protocols were developed aiming to reduce the time needed for differentiation and to increase the efficiency of differentiation. These objectives were pursued through the optimization of the concentrations, timing and sequence of administration of the small molecules used during the differentiation procedure *in vitro*. Notably, Du and colleagues developed a chemically defined differentiation strategy capable of generating highly enriched populations of motor neuron progenitors within approximately two weeks of differentiation. About 95% of them expressed the oligodendrocyte transcription factor 2 (OLIG2), which is expressed in the ventral neural tube and represents one of the earliest markers of the motor neuron lineage, during spinal cord development. These progenitors can be expanded for multiple passages while maintaining their developmental identity. Subsequently, they can be differentiated into mature cholinergic motor neurons co-expressing several transcription factors that confirm the achievement of the correct cell lineage. Among these, there are the canonical marker HB9, the insulin gene enhancer 1 and 2 (ISL1/2) members of the LIM-homeodomain proteins, and the choline acetyltransferase (ChAT) enzyme, which demonstrate the highest degree of maturation [65,66].

**Figure 2:**
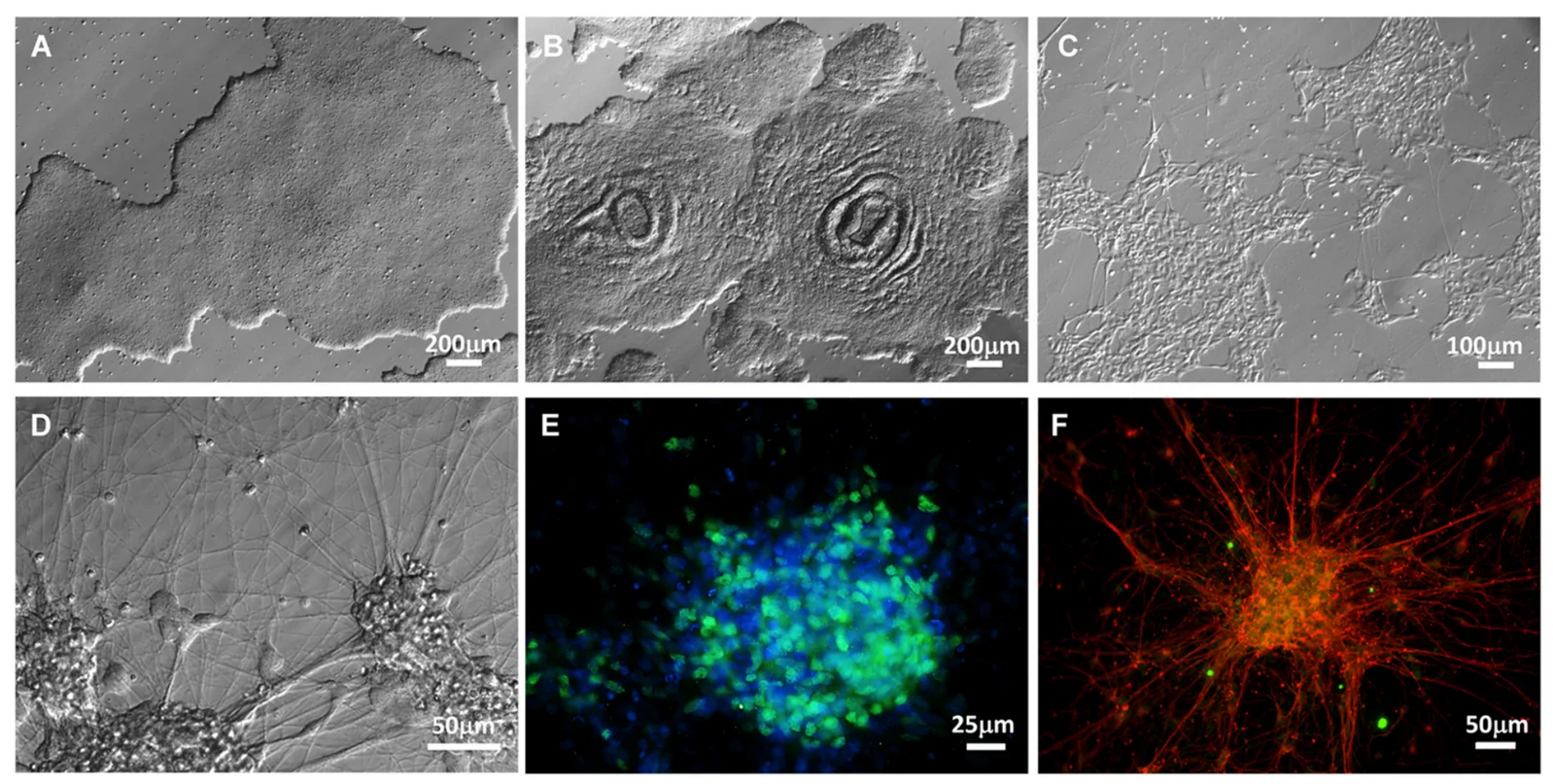
Stepwise differentiation of iPSCs into spinal motor neurons. Phase contrast images of iPSCs colonies derived from a non-neurological subject in the maintenance phase (**A**); neuroepithelial progenitor cells, following neural induction (**B**); motor neuron progenitors, after caudalization and ventralization (**C**); and mature spinal motor neurons (**D**). Immunofluorescence reveals that these cells express cell-specific markers, such as HB9 ((**E**) - HB9, green; Hoechst 33342, blue) and ChAT/βIII-Tubulin ((**F**)- ChAT, green; βIII-Tubulin, red).

To illustrate the progressive acquisition of spinal motor neuron identity, we took representative images at characteristic checkpoints during the differentiation protocol [67], which are shown in Figure 2. These images reveal the morphological transitions from undifferentiated iPSC colonies (Figure 2A) to neuroepithelial progenitors (NEPs) (Figure 2B), motor neuron progenitors (MNPs) (Figure 2C) and mature motor neurons (Figure 2D–F), reflecting the sequential developmental stages recapitulated *in vitro*.

Notably, we recently demonstrated that the majority of spinal motor neurons, differentiated according to this protocol, display the electrophysiological fingerprint of mature cells [67]. This amount of evidence indicates that the protocol described above can enable the differentiation of iPSCs from heterogeneous patient populations into spinal motor neurons, thereby making it possible to develop personalized approaches for potential new treatments. Furthermore, patient-derived iPSC technologies are crucial for both advancing *in vitro* ALS modelling and recapitulating the disease phenotype. The latter is particularly relevant in the context of sALS, which currently lacks reliable disease model.

Consistent with this need, an iPSC library from 100 patients with sALS was recently generated and used to conduct a population-wide phenotypic screening, thus detecting significant differences between iPSC-derived motor neurons obtained from healthy individuals and patients. The latter showed reduced survival and faster neurite degeneration, which correlated with the donor survival time. From a molecular standpoint, the transcriptional profile showed notable variations in the gene expression between diseased and control motor neurons [68]. This important result is consistent with the transcriptional analysis conducted on post-mortem sALS spinal cords [68], demonstrating once again that spinal motor neurons derived from iPSCs recapitulate the pathological process taking place in ALS patients.

Furthermore, since spinal motor neurons display different vulnerability to degeneration along disease progression [69], efforts are underway to refine differentiation protocols with the aim of enriching for the highly vulnerable neurons, such as those innervating the phrenic nerve [70], or highly resistant motor neuron subpopulations, including the oculomotor neurons [71]. 

### 3.2. Modelling UMNs

Several lines of evidence suggest that highly pure spinal motor neuron cultures represent a simplified system for modelling the disease, as they do not reproduce the overall complexity of the nervous system, which includes also the dysfunction of UMNs as well as the important contributions of the surrounding non-neuronal cells. Also known as Betz cells, UMNs are a small population of large pyramidal glutamatergic neurons located in the layer V of the motor cortex [72]. Intriguingly, a recent single-cell RNAseq study has revealed that their transcriptome is virtually indistinguishable from that of von Economo neurons (VENs), i.e., cells located in layer V of the prefrontal cortex, which are the main target of the degenerating process in Frontotemporal Dementia (FTD) [73]. In spite of the tremendous significance that would result from the ability to faithfully generate these cells *in vitro*, a protocol suitable to guide iPSCs into UMNs or VENs is still lacking, most likely because of the challenges to recapitulate *in vitro* the complex cascade of signaling pathways driving their specification during development [65]. Nevertheless, a recent study has shown the direct reprogramming of glial precursors cells into cortical motor neurons in mice by the simultaneous manipulation of the expression of distinct transcription factors [74]. Although Betz cells are considerably different from mouse cortical motor neurons [73], we speculate that important lessons can be learned from this new evidence and we envision that this combination of transcription factors will be soon tested to differentiate iPSCs into UMNs.

On the basis of this, present studies aiming at interrogating cortical involvement in ALS settle for generic glutamatergic neurons. To generate these cells, two sets of protocols are currently available: (i) those that rely only on the administration of small molecules to drive the differentiation, and (ii) those that involve the overexpression of the transcription factor Neurogenin2 [75]. By using this second approach, different groups have recently demonstrated that ALS neurons display dysregulation in gene expression and splicing, which affects cytoskeletal remodeling and synaptic function, as well as electrophysiological alterations [76,77].

## 4. Beyond Motor Neurons: ALS as a Multicellular Disorders

For many years, motor neuron degeneration has been considered the primary pathogenic event in ALS. However, later observations in animal models demonstrated that disease progression cannot be explained only by intrinsic motor neuron derangements. This pathology has been recognized as a multicellular disorder involving complex interactions between motor neurons and the neighboring non-neuronal cell populations. The first experimental evidence supporting this concept originated from studies in mutant *SOD1* ALS mouse models. Clement and colleagues [78] demonstrated that, in chimeric mice (consisting of mixtures of normal and mutant *SOD1*–expressing cells), mutant *SOD1*-expressing non-neuronal cells were able to cause damage to wild-type motor neurons. Conversely, normal non-neuronal cells significantly delayed degeneration of mutant *SOD1*-expressing motor neurons and critically influenced disease progression, thereby mimicking some aspects of ALS. This groundbreaking discovery instigated a shift in the pathogenetic hypotheses of ALS, from a cell-autonomous perspective (intrinsic pathogenetic mechanisms in motor neurons) to a non-cell-autonomous vision, involving non-neuronal cells, particularly glial cells, in the neurodegenerative process. Consistent with this idea, selective genetic ablation of *SOD1* mutants within microglia or astrocytes in transgenic animal models revealed that both glial cell types play an active role in ALS progression [79,80]. In addition, maladaptive oligodendrocyte maturation and functions were also reported in different animal models of the disease [81,82,83,84,85].

Notably, both ALS patients and rodent models were reported to exhibit strong activation of microglia and astrocytes, an event that gives rise to a process of "neuroinflammation", characterized by the chronic release of pro-inflammatory mediators [86]. The presence of activated microglia was confirmed by means of histopathological analyses of post-mortem tissues [87] as well as by exploiting imaging techniques, such as positron emission tomography (PET). In the SOD1 (G93A) mouse model of ALS, PET imaging studies using ligands of the mitochondrial 18kDa Translocator Protein (TSPO), a marker of microglial activation, revealed an increase in TSPO uptake in the brainstem and spinal cord, which was associated with a higher presence of activated microglia in regions affected by motor neuron degeneration [88].These findings were further confirmed by PET imaging in humans, where increased microglial activation was detected in the motor cortex of patients with *SOD1*-linked ALS, including pre-symptomatic carriers, thereby suggesting that neuroinflammation is an early event in the course of the disease [89]. Overall, these studies supported the involvement of microglia-mediated neuroinflammation in ALS and suggested that microglial activation was correlated with an accelerated disease course, even though determining in patients whether these links were causal a consequence of the pathology remains challenging [90]. Concerning astrocytes, these cells are probably the most studied glial contributors to ALS. Astrocytes are the most abundant and versatile glial cell population in the CNS [91], and perform a wide range of homeostatic functions that are essential for neuronal performance and survival. Over the years, the traditional vision of astrocytes has profoundly changed, moving from considering them as mere supportive cells for neurons to viewing them as active and dynamic partners. Astrocytes are, indeed, involved in an intricate intercellular network with neurons, blood vessels and other glial cell types. This places them in a strategic position to carry out a broad spectrum of functions, including extracellular potassium (K^+^) buffering, glutamate clearance through excitatory amino acid transporters 1/2 (EAAT1 and EAAT2), metabolic supply determined by a lactate shuttle from astrocyte to neuron, synaptic maturation and pruning, blood–brain barrier (BBB) sustenance, antioxidant defense, and neurovascular coupling through either vasodilating or vasoconstricting signals. Furthermore, even though astrocytes do not generate action potentials, they were described to exhibit a peculiar form of excitability based on fluctuations in intracellular calcium (Ca^2+^) concentrations. This activity is crucial for carrying out important functions of astrocytes, such as calcium-dependent gliotransmitter release, metabolic support, and communication with neurons and the neurovascular units. Consequently, dysregulation of this signaling pathway can profoundly disrupt both CNS homeostasis and intercellular communication, likely contributing to the development of neurodegeneration [92]. 

Beyond their physiological functions, astrocytes show remarkable structural and morphological changes in response to CNS injury and disease. Under pathological conditions, resting astrocytes undergo a series of morphological, molecular and functional alterations, which make them “reactive”. Morphologically, reactive astrocytes become hypertrophic and show an enlargement of their soma, thickening and elongation of primary processes, and increased branching complexity. These structural changes are commonly accompanied by enhanced expression of intermediate filament proteins, particularly the glial fibrillary acidic protein (GFAP), as well as vimentin and nestin, which globally reflects extensive cytoskeletal remodeling [93,94]. Importantly, morphological remodeling is tightly coupled with profound functional alterations. In fact, in specific pathological conditions, reactive astrocytes were shown to progressively lose their capacity to maintain extracellular glutamate homeostasis through reduced expression of the glutamate transporter EAAT2 (GLT-1 in rodents) and to display altered K^+^ buffering, impaired metabolic coupling with neurons, dysregulated Ca^2+^ signaling, and increased secretion of pro-inflammatory cytokines and reactive oxygen species (ROS). These functional deficits transform astrocytes from homeostatic regulators into active contributors to neurodegeneration [95]. 

Even though the morphological and functional alterations of reactive astrocytes have been only partially established [93], their contribution to ALS pathogenesis and progression has remained controversial for many years. Initially, astrocyte activation was largely considered a secondary response to motor neuron degeneration. However, this interpretation was progressively challenged by experimental evidence. As mentioned above, studies *in vivo* in transgenic models of ALS demonstrated that astrocytes actively contribute to disease progression [80]. A further aspect of astrocyte reactivity that has been described both in the spinal cord of sALS patients and transgenic mouse models of the disease concerns the process of astrocyte atrophy/degeneration [96,97]. Loss of astrocytes seems to be a major destabilizing event for the functional homeostasis of the territorial domain covered by that cell, representing a key detrimental event for the cells present in its microenvironment (e.g., motor neurons).

Additional support to the concept that functional astrocytes are necessary for the preservation of motor neurons *in vivo* was provided by transplantation studies aimed at restoring a healthy astrocytic environment in the spinal cord of ALS rodents. Transplantation of wild-type rat glial-restricted precursors [98], human iPSC-derived glial-rich neural progenitors [99], or human ESC-derived astrocyte-committed glial precursors [100] was shown to ameliorate the phenotype of ALS animal models. These findings provided the first proof that correcting astrocyte dysfunction could open new perspectives for astrocyte-targeted therapeutic strategies.

Concomitant investigations by Nagai and colleagues provided the first direct evidence *in vitro* that mutant *SOD1*-expressing astrocytes possess neurotoxic properties. More in detail, rodent astrocytes expressing the mutant protein determined both the selective loss of primary spinal motor neurons and damage to embryonic mouse stem cell-derived motor neurons in co-cultures, whereas other neuronal subpopulations remained largely unaffected [101]. Importantly, in the same study, researchers observed that soluble factors released by mutant astrocytes were neurotoxic, independently of the direct interaction between astrocytes and motor neurons. Similar results were confirmed in other investigations using mutant *SOD1*-expressing astrocytes and ESC-derived motor neurons [102,103,104].

Considering that these mechanisms have been identified in experimental models of ALS carrying *SOD1* gene mutations, it is plausible to postulate that they may apply only to this familial form of ALS, but not to the sporadic form of the human disease. However, studies conducted using human astrocytes have discredited this hypothesis. 

Haidet-Phillips and colleagues isolated astrocytes from the post-mortem spinal cord of patients with both familial and sporadic ALS. They demonstrated that astrocyte-mediated neurotoxicity induced degeneration of healthy motor neurons *in vitro*, independent of the heterogeneous genetic forms underlying ALS [105]. Likewise, Meyer and colleagues converted fibroblasts, derived from ALS patients, into induced neuronal progenitor cells and subsequently differentiated them into astrocytes. Regardless of whether astrocytes originated from fibroblasts of patients with sALS or fALS, co-culturing them with motor neurons demonstrated a shared toxic mechanism [106]. 

Despite mutation-specific differences, additional studies using ALS patient iPSC-derived astrocytes consistently indicated that astrocyte dysfunction converges toward common pathogenic pathways that ultimately compromise motor neuron survival [107,108,109].

**Figure 3:**
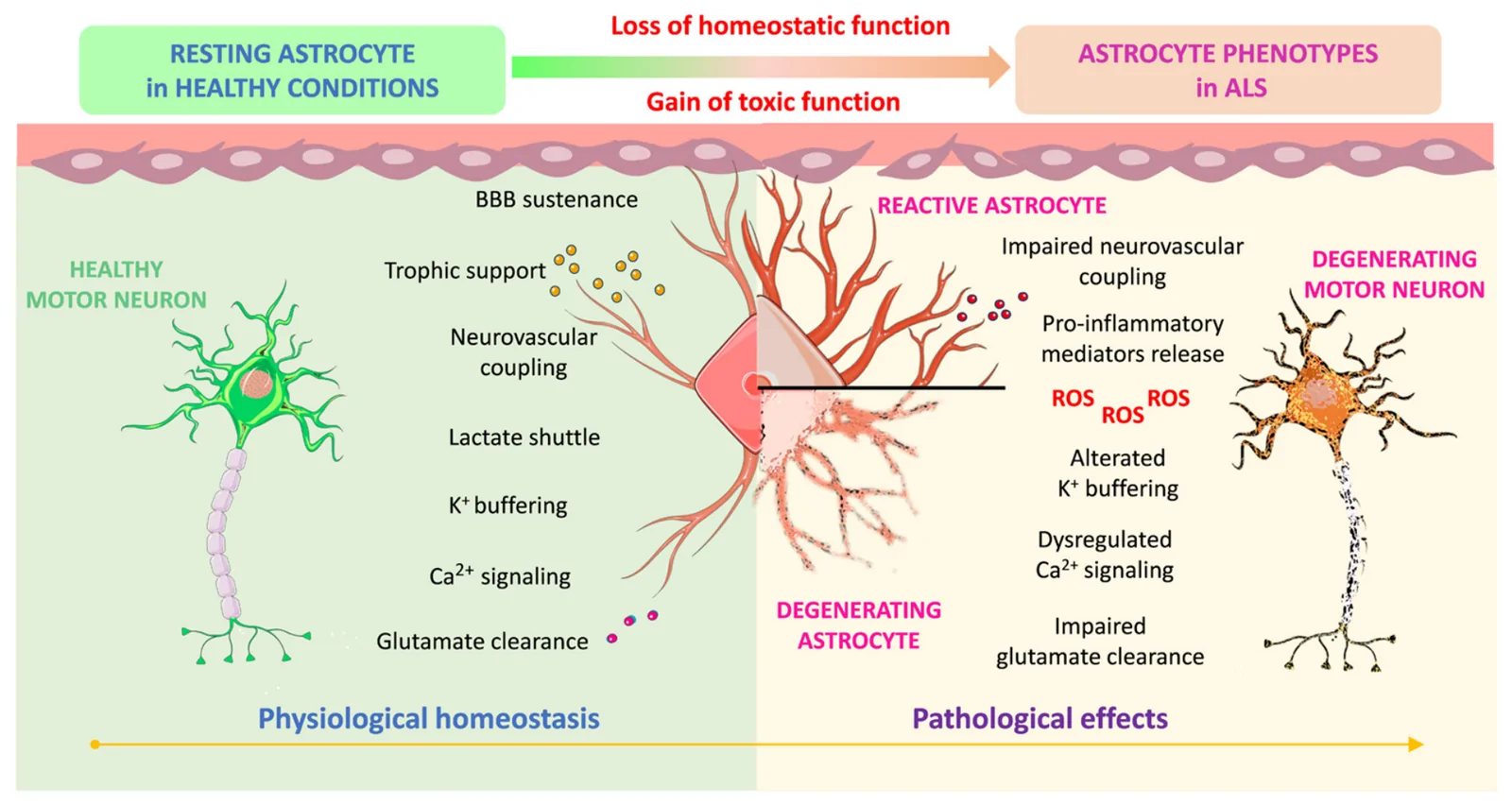
Physiopathological roles of astrocytes in healthy conditions and in ALS. Experimental evidence demonstrated multiple functions of astrocytes in physiological conditions and in the context of ALS. Astrocytic activities in healthy conditions are depicted in the left panel, whereas the right panel schematizes the two phenotypes (i.e., reactive and degenerating) as well as the functional deficits of astrocytes in ALS. Image adapted from Servier Medical Art (https://smart.servier.com/ accessed on 28 September 2026), licensed under CC BY 4.0 (https://creativecommons.org/licenses/by/4.0/ accessed on 28 September 2026) and modified using Microsoft PowerPoint 2019.

Together, these studies profoundly reshaped our understanding of ALS pathogenesis and progression, introducing the concept that the course of the disease is strongly influenced by mechanisms involving reciprocal interactions between motor neurons and glial cells, particularly astrocytes (Figure 3). At the same time, these discoveries highlighted an important limitation of conventional experimental models. Although animal studies were instrumental in identifying astrocyte-mediated neurotoxicity, species-specific differences in astrocyte biology limited their translational value, emphasizing the need for using human experimental systems capable of reproducing patient-specific astrocyte phenotypes. This challenge ultimately drove the development of human iPSC-based models, which currently represent one of the most powerful tools for investigating astrocyte biology in ALS.

### Human iPSC-Derived Astrocytes: A Tool to Fill the Gap Between Animal Models and Human Disease

The experimental evidence discussed above unequivocally established glial cells, particularly astrocytes, as active elements in ALS disease. Nevertheless, translating these discoveries into a comprehensive understanding of the human disease remains challenging. Importantly, compared with rodent astroglial cells, human astrocytes are larger, exhibit a higher degree of morphological complexity and contact a substantially greater number of synapses. Human astrocytes also show distinct transcriptional signatures and functional properties, including enhanced calcium signaling, increased metabolic specialization and a more elaborate regulation of synaptic activity [110]. These species-specific differences in the astrocytes substantially limit the ability of the animal models to faithfully recapitulate the complex cellular interactions that occur in the human CNS. 

Over the years, increasingly sophisticated human models have been established, evolving from primary astrocytes isolated from post-mortem human tissues, through ESC-derived astrocytes, to patient-specific iPSC-derived astrocytes. Each model has progressively overcome the limitations of its predecessor, achieving experimental systems capable of reproducing human astrocyte biology as closely as possible.

Regarding primary astrocytes isolated from post-mortem tissues, despite their biological relevance, this model rapidly revealed several drawbacks. Obtaining human astrocytes from tissues remains a challenge, and their molecular integrity may be compromised. Recent efforts have been made to standardize culturing conditions and protocols [111], but technical variability can still compound patient-associated differences, an event that restricts their use for large-scale mechanistic studies or drug screening approaches. On top of that, post-mortem astrocytes mirror the last stage of the disease. As a consequence, they provide only a static image of advanced neurodegeneration, making them unsuitable for investigating the earliest molecular events driving disease initiation. To overcome these limitations, researchers turned to human ESCs, which provided the first renewable source of human astrocytes. Unlike primary cultures, human ESC-derived astrocytes can be produced in virtually unlimited numbers and can be efficiently differentiated into transplantable neural precursors using defined culture conditions. This approach can generate highly enriched neural progenitor populations with robust neurogenic and gliogenic potential [112]. Furthermore, a differentiation protocol established by Krencik and colleagues enabled the differentiation and maintenance of functionally distinct astroglial subtypes by recapitulating key developmental signaling pathways. In this way, they produced astrocytes that exhibit molecular and functional characteristics of their *in vivo* counterparts [113]. Remarkably, these cellular models allowed researchers to overcome many of the limitations associated with primary astroglial cells derived from post-mortem tissues. Yet, human ESC-derived astrocytes were also found not to be completely suitable for modelling ALS. Since human ESC lines originate from healthy embryos, they lack the patient-specific genetic background that underlies disease susceptibility and, therefore, cannot fully reproduce the pathological phenotypes. Although genome editing can introduce individual ALS-associated mutations into embryonic stem cells, these models remain unable to capture the complex combination of genetic modifiers that characterize individual patients, particularly in the case of sALS [114]. 

At this stage, iPSC technology emerged as a powerful alternative, combining the strengths of previous cellular models while overcoming many of their limitations. Unlike cells from post-mortem tissues, human iPSCs allow astrocytes to be generated from living patients at different stages of disease progression. At the same time, in contrast to human ESC-derived astrocytes, they faithfully maintain both pathogenic mutations and individual genetic modifiers, making it possible to investigate mutation-specific and patient-specific disease mechanisms under controlled experimental conditions. For example, astrocytes generated from patient-derived iPSCs and carrying mutations in the *TARDBP* [107] or *VCP* [115] genes were shown to exhibit increased vulnerability, similar to the observations reported in mutant *SOD1* transgenic mice and sALS spinal cord tissues [96,97]. Furthermore, a study by Fujimori and colleagues leveraged iPSC technology to generate patient-specific stem cells and established a wide range of *in vitro* models of sALS [116]. These models revealed substantial phenotypic heterogeneity among patients, including differences in neuronal degeneration patterns, abnormal protein aggregation profiles, mechanisms of cell death, and the onset and progression of disease-related phenotypes *in vitro.* Based on these features, a case-clustering system was developed to classify heterogeneous sALS models according to their distinct cellular and molecular characteristics. In this framework, the “Answer ALS” project represented an important step towards the translational use of iPSC-derived motor neurons in neurodegenerative disease research. This is a project that has allowed the creation of an archive of approximately 1000 iPSC lines derived from ALS patients and healthy controls, according to a standardized protocol. The platform enabled reproducible comparison of disease-associated phenotypes and supported applications in ALS modelling, drug screening, and therapeutic discovery [117]. Furthermore, it contributed to the data repository “Neuromine” (https://dataportal.answerals.org/home accessed on 28 September 2026), which integrates cell culture and patient multi-omics datasets with detailed clinical information.

Although iPSC-derived astrocytes and motor neurons have considerably advanced our understanding of ALS, these studies have very recently been complemented by others focusing on microglia [118] and oligodendrocytes [119]. Importantly, studying each cellular population in isolation only partially reflects the complexity of the disease. Increasing evidence in fact indicates that motor neuron degeneration arises from continuous bidirectional communication between neurons and the surrounding glial cells [120]. Consequently, the development of co-culture systems combining human iPSC-derived motor neurons and glial cells, such as astrocytes, represented a natural evolution of *in vitro* disease modelling, allowing investigators to recreate the non-cell-autonomous interactions that characterize ALS. Compared with monocultures, co-culture systems preserve reciprocal signaling among different cell populations. Reconstituting these reciprocal interactions *in vitro* provides a substantially more physiological platform for investigating disease mechanisms than studying either population independently.

One of the earliest demonstrations of the potential of human co-culture models came from Zhao and colleagues, who demonstrated that astrocytes derived from patients carrying *C9orf72* repeat expansions impaired electrophysiological maturation and increased the vulnerability of healthy iPSC-derived motor neurons. These observations supported the existence of mutation-specific astrocytic phenotypes converging toward common neurodegenerative pathways [121]. Furthermore, in a different study, *FUS*-mutant iPSC-derived astrocytes were incorporated into a human motor unit microfluidics model, thus demonstrating that ALS astrocytes are cytotoxic and dampen the ability of motor neurons to establish functional junctions with myotubes [109]. 

Altogether, this amount of evidence suggests that, by combining healthy and ALS-derived motor neurons with either healthy or ALS-derived astrocytes in different experimental configurations, it is possible to directly determine which cellular compartment primarily drives specific pathological phenotypes. Beyond investigating disease mechanisms, co-culture systems have rapidly become valuable platforms for the identification of several therapeutic strategies aimed at restoring astrocytic homeostatic functions, reducing inflammatory signaling and improving motor neuron survival in patient-specific cellular contexts.

The study of Dennys et al. is such an example. In a co-culture system, researchers demonstrated an improvement in motor neuron survival after treatment with (SP-4-2)-[[2,2’-(1,2-dimethyl-1,2-ethanediylidene)bis[N-methylhydrazinecarbothioamidato-κN2,κS]](2-)]-copper (CuATSM), a compound able to reduce the toxicity exerted by ALS patient-derived astrocytes on human motor neurons [122].

Despite their considerable advantages, 2D co-culture systems still provide only a simplified representation of the human CNS. Astrocytes and motor neurons grown on flat substrates lack the 3D extracellular matrix, mechanical properties and multicellular organization of nervous tissue. Moreover, additional cellular populations—including microglia, oligodendrocytes, endothelial cells and pericytes—remain largely absent, preventing the reconstruction of the full neurovascular and neuroimmune environments that contribute to ALS progression. These limitations have stimulated the development of increasingly sophisticated 3D culture systems, including organoids, which aim to reproduce more faithfully the tissue architecture and cellular diversity.

## 5. Increasing Model Complexity: Organoids and Organ-on-a-Chip Models of ALS Using iPSCs

Motor neurons, astrocytes, oligodendrocytes, microglia and interneurons are organized *in vivo* within a highly specialized 3D microenvironment, where continuous cell–cell interactions, extracellular matrix components and spatial gradients of signaling molecules collectively regulate tissue homeostasis. These architectural features are largely absent in conventional 2D cultures, limiting their ability to realistically reproduce the complexity of ALS pathology. To bypass these limitations, increasing attention has been directed towards the development of 3D culture systems, particularly organoids generated from human iPSCs [123].

### 5.1. Region-Specific Organoids as Tools for Mechanistic Investigations and Drug-Discovery in MNDs

In recent years, different groups have reported that both cortical and spinal cord organoids recapitulate several ALS-associated phenotypes, including impaired neuronal maturation, altered electrophysiological network activity, reactive gliosis, and progressive motor neuron degeneration (reviewed in [124,125]). To strengthen the relevance of these early observations, it soon emerged the need for investigating more accurate models. Specifically, global efforts have been devoted to obtaining organoids consisting of the precise cell composition of the CNS area of interest. In the context of ALS, this implies that the presence of the specific cell types involved in the disease should be investigated in the resulting organoids. Cells of interest include UMNs in brain organoids, LMNs in spinal organoids, as well as different glial cell populations in both model types. Interestingly, the molecular fingerprint of cells derived from cerebral organoids in the first 3 months of culture was reported to correspond to primary cells in the first trimester of development, whereas organoids around 3 months of culture or more match the transcriptome of second-trimester primary cells [126]. This body of evidence suggests that the results obtained from the investigations into 3D cultures should be evaluated in light of their time in culture, their composition and their relative immaturity, a limitation that is shared with 2D cultures (see paragraph 1.3 above).

The importance of the time in culture was further confirmed by scRNAseq studies on cortical organoids, which unraveled the emergence in the long term of deep-layer glutamatergic neurons expressing markers of corticofugal projection neurons [127,128]. 

A key limitation of CNS organoid protocols is that they guide cells into the neuroectoderm lineage by inhibiting mesoderm and endoderm formation. This implies that cerebral organoids lack the complete set of cell types present in the brain, particularly microglia. These observations prompted the revision of the procedures to produce cerebral organoids by reducing the levels of heparin (a neuroectoderm stimulant) and delaying basement membrane matrix embedding of the organoids [129]. An alternative approach to obtain microglia-containing organoids is based on the differentiation of microglial cells from iPSCs and their subsequent integration into pre-established guided cortical organoids, thus gaining a region-specific molecular signature [130,131,132,133].

In parallel studies, spinal cord organoids were also developed. Early protocols enabled the time-dependent generation of diverse spinal cord cell types, including motor neurons, as revealed by the expression of specific markers, such as ISL1, ChAT and Forkhead box protein P1 (FOXP1) [134]. In addition, the expression of markers of the rostro-caudal axis revealed that some cells had a cervical identity, while the majority had a brachial/thoracic identity. Conversely, the expression of markers of lumbar motor neurons remained undetectable [134]. This limitation was recently overcome by the development of a refined protocol that allowed the generation of organoids that included cells with lumbar identity [135]. The presence and characterization of motor neurons in mature spinal organoids have been further confirmed through transcriptomic, molecular and functional approaches, including electrophysiological recordings, calcium imaging, and multielectrode array analyses [136,137]. Although the cellular composition of spinal cord organoids has been recently rectified by adding microglial cells [138,139], these implementations have not yet been extended to ALS models. 

In the context of motor neuron disorders, neural organoids have provided valuable models to investigate some disease-associated pathogenic mechanisms. For example, cortical organoids derived from ALS-*C9orf72* patients have been exploited as a platform for investigating preclinical aspects of ALS/FTD as well as for exploring new potential therapeutic approaches. These findings revealed early-stage dysfunctions of the ALS phenotype, including DNA damage and impaired proteostasis in both neurons and astrocytes, defects that could be resolved by dampening endoplasmic reticulum stress [140]. Furthermore, microglia activation was detected in unguided cerebral organoids of different ALS subtypes when these structures were generated with a microglia-permissive protocol [141,142]. More recently, Casiraghi et al. demonstrated that brain organoids undergoing chronic oxidative stress, induced by exposure to sodium arsenite, develop cellular defects resembling those observed in ALS-*C9orf72*. Moreover, the administration of rapamycin, an autophagy inducer, mitigated these phenotypes [143]. 

In parallel investigations, ventral spinal organoids derived from SMA patients were shown to recapitulate the rapid motor neuron degeneration typical of the disease [134,144]. Furthermore, in ALS-associated lines, spinal organoids led to the identification of alterations in the Linker of Nucleoskeleton and Cytoskeleton (LINC) complex, which may translate into a possible biomarker and/or a potential target for drug discovery [145]. Taken together, these studies demonstrate the ability of organoids to recapitulate key elements of MND pathology and, consequently, suggest that they can be used for drug discovery applications. However, they also highlight the relevance of choosing the most appropriate disease model as well as the need for constant refinement of experimental protocols as prerequisites for the successful outcome of therapeutic development.

### 5.2. Model Refinements: Assembloids and Organ-on-Chip Models as Tools to Investigate Functional Interactions 

A further advancement of the modelling approach discussed so far is represented by assembloids, which are generated through the fusion of two or more organoids. While organoids can be regionalized to develop characteristics of a specific brain or spinal cord region, assembloids enable the reconstruction of the interactions between distinct neural compartments. This approach allows the study of functional connectivity between different regions of the nervous system and provides an opportunity to investigate how their interactions are altered under pathological conditions. In this context, a tremendous technical advancement came from the development of cortico-motor assembloids, which combine cortical with spinal and muscular organoids to model the entire descending motor pathway [124,127,146].

Despite its significant advantages, organoid technology still presents several important limitations. In addition to the high costs, reproducibility and standardization remain challenges. Furthermore, the absence of a functional vascular system limits the diffusion of oxygen and nutrients to the organoid core, interrupting the maturation of the latter and causing culture disruption [147]. To overcome these limitations, an interesting opportunity arose from slicing cortical organoids for long-term culturing at the air-liquid interface, a strategy which allowed modelling disease-relevant phenotypes in ALS [140].

These restrictions have pushed forward the technologies for the development of microfluidic organ-on-a-chip systems, which integrate human stem cell-derived neural populations within bioengineered devices capable of reproducing tissue architecture, while simultaneously controlling fluid flow, biochemical gradients and mechanical stimuli. These features made these devices more physiologically relevant than the previous ones. Thus, it appears that these extremely versatile systems can be exploited for studying physiological or pathological processes as well as for the development and testing of drugs, in various fields [148]. 

In the context of ALS, micro-physiological systems of human BBB-chip have been developed with iPSC-derived brain microvascular endothelial-like cells, astrocytes, and neurons. The resulting monolayer expressed specific markers of cerebral vascularization, somehow exceeding one of the limits of organoids. This system realistically reproduced the structure and function of the BBB, allowing investigations of the mechanisms underlying neurological diseases and the transport of drugs across the BBB [149]. More recently, Lall and colleagues developed a microfluidic device named spinal cord chip (SC-chip) to model sALS. The peculiar feature of this device is that it combines iPSC-derived motor neurons (from ALS patients) with brain microvascular endothelial-like cells. The model revealed ALS-specific alterations in neurofilament expression and dysregulation of glutamatergic and synaptic signaling. Furthermore, single-nuclei RNA sequencing (snRNAseq) allowed to identifying two distinct motor neurons subpopulations. Both of these expressed the canonical motor neuron markers ISL1 and ChAT. However, one population was typified by MNX1 expression, consistent with a skeletal motor neuron identity, while the other by Paired Like Homeobox 2B *(PHOX2B)* expression, a marker of cranial motor neurons [17].

Additional organ-on-chip systems enabled the generation of models aiming at mimicking the descending motor pathway. For example, Charalampopoulou et al. recently developed a protocol to record the electrophysiological properties of connected corticofugal glutamatergic neurons and spinal cord motor neurons in the presence of region-specific astrocytes [150], a powerful approach that will likely be applied to ALS models in the near future. Furthermore, in a microfluidic system, iPSC-derived skeletal muscle bundles were integrated with iPSC-derived spinal motor neurons from a sALS patient, resulting in an ALS motor unit on-a-chip model. Combined with optogenetic technology, this system allowed the comparative assessment of motor neuron activity and muscle contraction in ALS and non-ALS motor units. Intriguingly, rapamycin and bosutinib (a Src/cAbl kinase inhibitor) co-treatment was shown to partially restore muscle contraction under ALS conditions [151]. 

**Figure 4:**
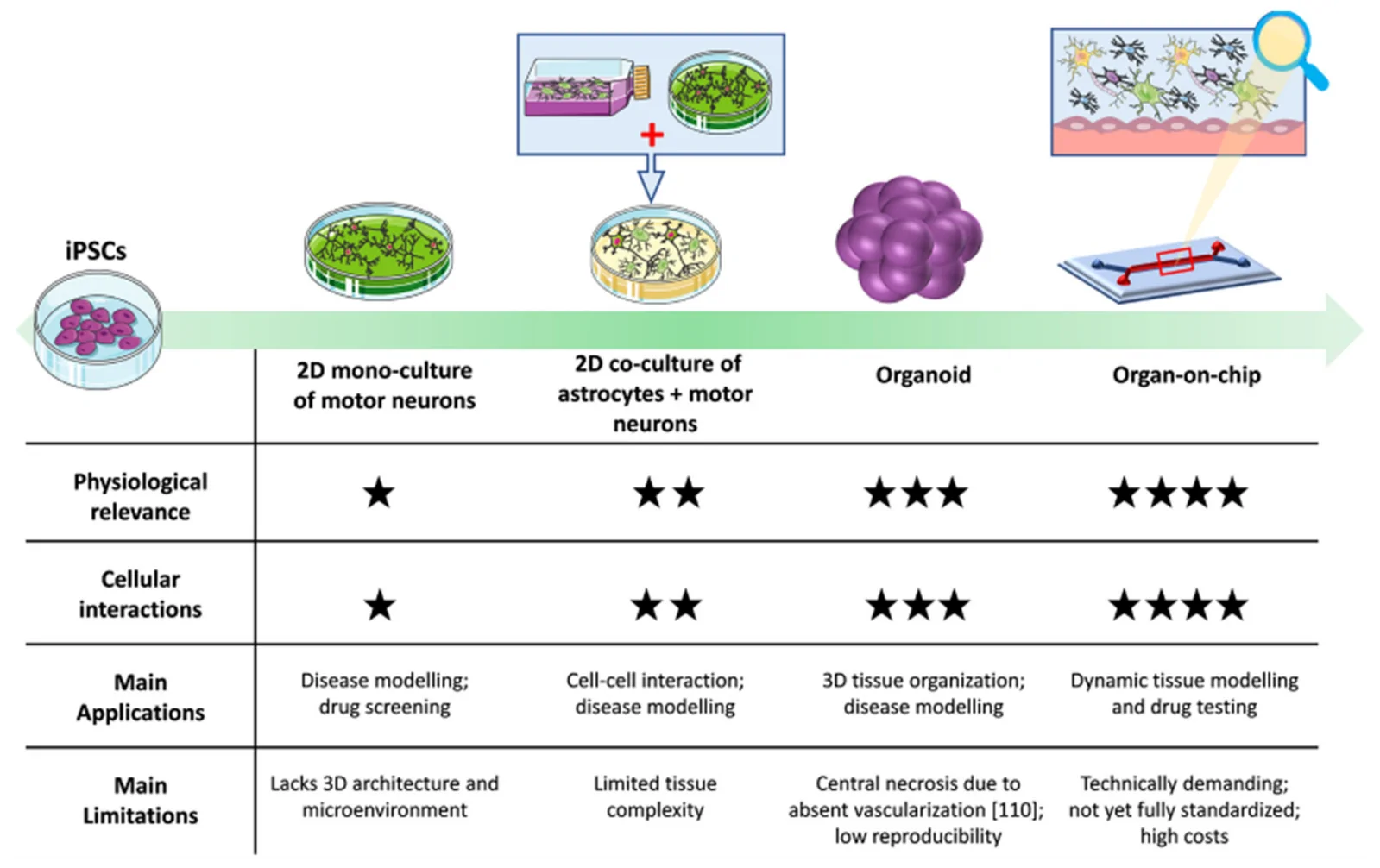
Evolution of the *in vitro* models: structural differences, main applications and biological limitations. The green arrow, from left to right, indicates increasing biological complexity and physiological relevance across iPSC-derived *in vitro* models, highlighting progressively advanced applications while introducing greater technical challenges. Image adapted from Servier Medical Art (https://smart.servier.com/ accessed on 28 September 2026), licensed under CC BY 4.0 (https://creativecommons.org/licenses/by/4.0/ accessed on 28 September 2026) and modified using Microsoft Power Point 2019.

Collectively, the evolution of these experimental platforms has substantially expanded our ability to model the complexity of ALS *in vitro*. Although each model presents intrinsic strengths and limitations, together they provide complementary tools that allow the investigation of disease mechanisms at progressively higher levels of biological complexity. This means that 2D cultures, co-culture systems, organoids and organ-on-a-chip technologies represent complementary approaches rather than alternative ones (Figure 4). Each of them addresses distinct biological questions while contributing to a more comprehensive reconstruction of ALS in vitro.

## 6. Concluding Remarks and Future Directions

As highlighted throughout this review, the extraordinary progress achieved over the past two decades through the use of iPSCs has profoundly transformed the landscape of biomedical research. As a testament to the advancements achieved with *in vitro* models, the FDA Modernization Act 2.0 and 3.0 pave the way for the complete replacement of animals for preclinical testing of new drugs [152]. 

In the field of ALS, the development of human stem cell-based models has progressed in parallel with the evolution of our understanding of disease pathogenesis.

Early *in vitro* studies primarily focused on motor neurons, reflecting the view that ALS was exclusively a cell-autonomous disease. As subsequent evidence demonstrated the pivotal contribution of astrocytes and other glial cell populations to disease progression, experimental models progressively evolved to incorporate these additional cellular components. This led first to the development of 2D co-culture systems and subsequently to more sophisticated 3D models, including organoids and bioengineered organ-on-a-chip platforms, which aim to recapitulate increasingly faithful *in vitro* representations of the motoneuronal biological microenvironment. 

Nevertheless, each of these experimental approaches remains valuable for investigating distinct aspects of disease biology. While iPSC-derived monocultures are indispensable for dissecting cell-autonomous mechanisms under highly controlled conditions, iPSC-derived co-culture systems have allowed the study of the bidirectional crosstalk between motor neurons and glial cells, particularly astrocytes. Organoids have introduced an additional level of structural organization and multicellular architecture, whereas organ-on-a-chip platforms have combined these advantages with precise control over fluid dynamics, biochemical gradients, and mechanical stimuli. Rather than representing competing approaches, these platforms should therefore be regarded as complementary experimental tools, each capturing specific aspects of ALS neurobiology.

Remarkably, the use of iPSC-derived motor neurons has already led to the identification of therapeutic candidates (reviewed in [153]). In keeping with this, the antiepileptic drug retigabine was shown to reduce spinal motor neuron hyperexcitability and degeneration observed in cellular models of familial ALS with distinct genetic lesions [154,155]. Supported by this evidence, a clinical trial was soon undertaken. Although its results demonstrated that retigabine is able to mitigate hyperexcitability in ALS patients [156], no further follow-up study has been reported to date. In addition, Fujimori et al. identified ropinirole as a drug candidate in various clinical cases of ALS. Interestingly, this drug showed protective effects in iPSC-derived cells modelling different patient subpopulations, including not only patients with genetic forms of the disease (*FUS* and *TARDBP*) but also sALS patients [116]. A few years later, ropinirole entered a phase 1/2a clinical trial for ALS, showing promising results in terms of safety and its ability to delay the decline of motor function [157]. Another candidate under development is bosutinib. First identified as a neuroprotective agent in a phenotypic screening of ALS-derived spinal motor neurons [158], this compound was also shown to ameliorate motor dysfunction in organ-on-chip models [151]. These encouraging results expedited the translation to a phase 1 clinical trial, which revealed an acceptable safety profile [159]. This evidence prompted the registration of a phase 2 trial [160], whose results have not yet been released. 

More recently, the development of a sophisticated platform for thorough phenotypic characterization allowed for the first time the reproduction of key features of ALS, including patient-related variability in disease progression and response to riluzole [68]. Taking advantage of this tool, an extremely promising combination of drugs (which includes riluzole) with synergistic neuroprotective properties was identified, and its benefit to motor neurons was also shown on lines obtained from patients non-responding to monotherapy [68], thereby demonstrating how iPSC-derived cells could become instrumental for patient stratification and personalized medicine.

Despite these significant advances, important challenges remain. Current models still fail to fully recapitulate several key features of the adult spinal cord, including vascularization, long-term aging, systemic immune interactions, and the remarkable cellular heterogeneity that characterizes the human CNS. Furthermore, improving the reproducibility and standardization of differentiation protocols remains essential for translating these technologies into robust platforms for drug discovery and precision medicine. We envision that the implementation of automated systems and artificial intelligence tools will expedite all these steps to enable iPSC-derived models to achieve their full potential (reviewed in [161]). Finally, reconstructing motor neuron diseases "in a dish" no longer means generating human motor neurons. Rather, it involves recreating the intricate cellular ecosystem in which neurodegeneration occurs and develops. This conceptual shift has fundamentally modified the way ALS is investigated and provides a solid foundation for the development of more effective therapeutic strategies, increasingly aligned with the principles of personalized medicine. In line with this, refining our knowledge of iPSC-derived cell biology will likely increase the safety and the efficacy of neural cell transplants, thus complementing the actual therapeutic portfolio in synergy with pharmacological, gene-therapy and rehabilitation programs. 

## Figures and Tables

**Figure A1:**
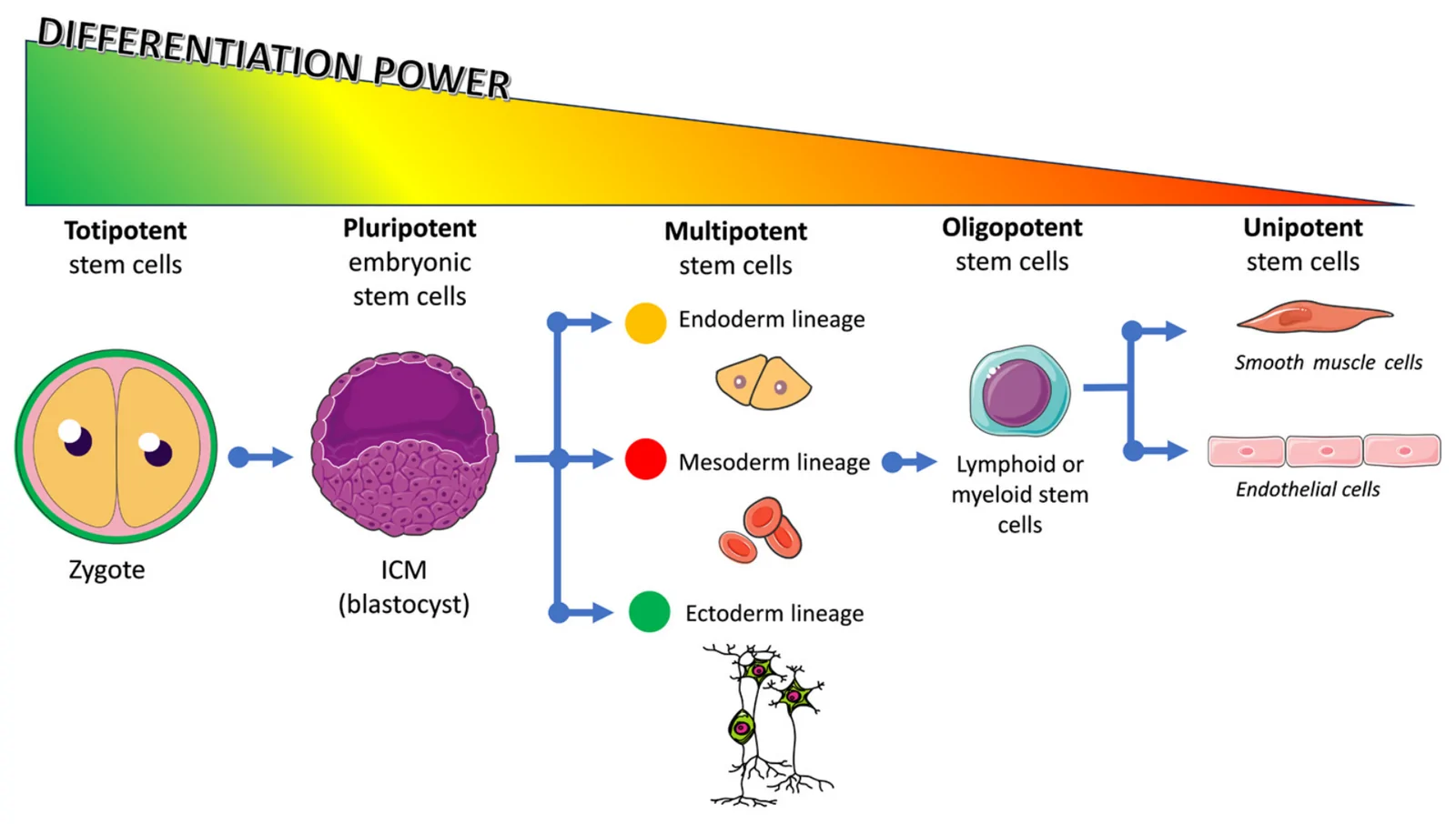
Schematic representation of the SC differentiation potential. Stemness levels allow categorizing cells by their differentiation capacity, from totipotency of the zygote to pluripotency of the inner cell mass (ICM) inside the blastocyst, followed by differentiation into multipotent stem cells, i.e., cells that can generate cell lineages, usually within their specific germinal layer (endoderm, mesoderm, ectoderm). From the mesoderm layer originate oligopotent stem cells, which are capable of differentiating into unipotent stem cells, such as smooth muscle cells or endothelial cells. Image adapted from Servier Medical Art (https://smart.servier.com/ accessed on 28 September 2026), licensed under CC BY 4.0 (https://creativecommons.org/licenses/by/4.0/ accessed on 28 September 2026) and modified using Microsoft PowerPoint 2019.

## Data Availability

No new data were created or analyzed in this study. Data sharing is not applicable to this article.

## Abbreviations

The following abbreviations are used in this manuscript:

iPSCs	Induced Pluripotent Stem Cells
ALS	Amyotrophic Lateral Sclerosis
SCs	Stem Cells
ESCs	Embryonic Stem Cells
Oct4	Octamer-binding Transcription factor 4
Sox2	Sex-Determining Region Y-Box transcription factor 2
Klf4	Krüppel-like factor 4
CNS	Central Nervous System
PD	Parkinson’s Disease
3D	Three-Dimensional
sc/snRNAseq	Single-cell/single-nucleus RNA-sequencing
2D	Two-Dimensional
AGIIR	Genome Induced Immune Response
CRISPR	Clustered Regularly Interspaced Short Palindromic Repeats
Cas	CRISPR-associated protein
MNDs	Motor Neuron Diseases
UMNs	Upper Motor Neurons
LMNs	Lower Motor Neurons
PLS	Primary Lateral Sclerosis
PMA	Progressive Muscular Atrophy
SMA	Spinal Muscular Atrophy
sALS	Sporadic Amyotrophic Lateral Sclerosis
fALS	Familial Amyotrophic Lateral Sclerosis
SOD1	Superoxide Dismutase 1
C9ORF72	Chromosome 9 Open Reading Frame 72
TARDBP	Trans-Activation Response DNA Binding Protein
TDP-43	TAR DNA Binding Protein 43
FUS	Fused in Sarcoma
VCP	Valosin-Containing Protein
HB9	Homeobox 9
MNX1	Motor Neuron and pancreas Homeobox 1
RA	Retinoic Acid
SHH	Sonic Hedgehog
OLIG2	Oligodendrocyte Transcription Factor 2
ISL1/2	Insulin Gene Enhancer 1 and 2
ChAT	Choline Acetyltransferase
NEPs	Neural Progenitors
MNPs	Motor Neuron Progenitors
VEN	von Economo Neuron
FTD	Frontotemporal Dementia
PET	Positron Emission Tomography
TSPO	Translocator Protein
K^+^	Potassium
EAAT1/2	Excitatory Amino Acid Transporters 1 and 2
BBB	Blood–Brain Barrier
Ca^2+^	Calcium
GFAP	Glial Fibrillary Acidic Protein
GLT-1	Glutamate Transporter 1
ROS	Reactive Oxygen Species
CuATSM	(SP-4-2)-[[2,2’-(1,2-dimethyl-1,2-ethanediylidene)bis[N-methylhydrazinecarbothioamidato-κN2,κS]](2-)]-copper
FOXP1	*Forkhead box protein P1*
LINC	Linker of Nucleoskeleton and Cytoskeleton
SC-chip	Spinal cord chip
PHOX2B	*Paired-Like Homeobox 2B*
ASCs	Adult Stem Cells
ICM	Inner Cell Mass

## Appendix A. Highlights on Stem Cells

The extraordinary power of SCs lies in their undifferentiated state, which is characterized by two pivotal features: (i) the capacity to self-renew indefinitely and (ii) the potential to differentiate into diverse cell types [162]. From the earliest stages of embryogenesis through adulthood, SCs orchestrate tissue specification, organ formation and repair of specific tissues and organs through highly coordinated molecular mechanisms involving transcriptional regulation, epigenetic remodeling, metabolic adaptation and extracellular signaling pathways [163]. Stem cells can be classified according to both their origin and their differentiation potential. Based on their origin, SCs may be embryonic (fetal) or somatic (adult). According to their differentiation potential, they are classified into totipotent, pluripotent, oligopotent, multipotent and unipotent categories, a continuum that reflects a progressive restriction of their capacity to differentiate into specific and distinct cell lineages (Figure A1) [164].

Totipotency indicates the highest degree of differentiation potential. It is constrained to a brief period, limited to the earliest days of embryonic development. A totipotent cell is, for example, the zygote that can evolve into a new organism, thus giving rise to the embryo and extra-embryonic structures [165]. As embryonic development progresses, the zygote divides and generates the blastocyst, which presents two distinct cell groups, i.e., the trophectoderm and the inner cell mass (ICM). The trophectoderm develops into the placenta, while the stem cells within the ICM, which are pluripotent, evolve to form the fetus. Notably, this pluripotent lineage has a lower potency compared to totipotent cells, which can differentiate into all body cell types [22]. Pluripotent stem cells can in fact generate multiple lineages from the three primary germinal cell layers (endoderm, mesoderm, and ectoderm), but they cannot develop into extra-embryonic structures, such as the placenta. ESCs, initially isolated from a mouse ICM [166] and subsequently from the human blastocyst ICM [167], retain characteristics of pluripotency [165]. Prior to becoming crucial tools in regenerative medicine, ESCs represented the first experimental model to study the molecular mechanisms that regulate embryonic development. By serving as sophisticated models in preclinical testing, ESCs further emerged as essential tools to verify the efficacy and safety of new drugs. This multifaceted potential not only emphasized the importance of ESCs to address biological questions but also contributed to accelerating scientific research, redefining the future of medical therapies [164]. Despite the fact that human ESCs have been extensively employed in various contexts, their use is subject to certain limitations. Among others, ethical restrictions pose significant challenges, as the isolation of ICM cells involves the destruction of a blastocyst, i.e., an early-stage human pre-implantation embryo. As a consequence, many countries have imposed strict laws or total bans on research involving the isolation of human ESCs. Further limitations include the risk of teratoma formation and immunological rejection upon ESC transplantation. Finally, practical difficulties are also raised by the hurdle of guiding cell specialization *in vitro* to obtain only the desired cell type [21,168].

As pluripotent stem cells undergo specialization, their differentiation potential progressively decreases, leaving space for multipotent, oligopotent and unipotent potency. Multipotent stem cells can differentiate into multiple specialized cell types, although their potential is typically restricted to a specific cellular lineage derived from one of the three primary germinal layers. Among others, this category of cells also includes Adult Stem Cells (ASCs). Throughout adult life, ASCs are responsible for the maintenance of tissue homeostasis. They are located within specialized microenvironments, called stem cell niches, where they receive stimuli that determine their fate. ASCs can remain in a quiescent state indefinitely. Alternatively, under specific conditions, they can start proliferating and differentiating to replace exhausted or dead cells in response to tissue damage caused by injury or disease [169].

Oligopotent stem cells possess a more limited differentiation capacity compared to multipotent stem cells and they can generate only a few specific cell types. Examples of oligopotent cells are lymphoid or myeloid stem cells. Unipotent cells can give rise to a single specific cell type, as they possess the most limited differentiation capacity [170].

## Appendix B. Methods to Induce Pluripotency

Current research in the field of iPSC generation is progressing along two parallel lines of investigation: on the one hand, different methods have been developed over the years to deliver OSKM factors into somatic cells and to induce pluripotency; on the other hand, attempts have been made to achieve the same results by exploiting small molecules instead of genetic manipulation. The first research area was built on traditional methods, conveying exogenous reprogramming factors via viral transduction or non-viral delivery. In both cases, there are two possible options, i.e., expression as a result of transcription factor-encoding DNA integration into the host cell genome (integrative methods) or transient expression (non-integrative methods). More specifically, the delivery of reprogramming factors by lenti/retroviruses leads to their integration into the genome and, thus, to their stable expression for iPSC induction [3,171]. Yet, this approach may pose the risk of insertional mutagenesis and cause incomplete silencing or residual expression of reprogramming factors. At variance with this, the non-integrating approach is based on the use of alternative viral vectors, such as adenovirus, adeno-associated virus, or Sendai virus [172,173,174,175]. The advantage of this strategy is that non-integrating viral vectors are gradually cleared from proliferating iPSCs, resulting in reprogramming without permanent OSKM integration. In addition, non-viral reprogramming methods include various strategies based on the use of transposons [176,177], episomal plasmids [178,179], mRNA [180,181], synthetic self-replicative RNA [182], miRNA [183,184,185], minicircle DNA [186], and CRISPR activation [187,188]. Remarkably, non-integrative episomal vector methods have a better safety profile compared with integrative methods using viruses. Thus, episomal plasmids are commonly used to generate iPSCs for clinical applications. A further advantage of this strategy is its low cost compared with that associated with the use of Sendai virus, combined with no reduction in reprogramming efficiency.

The limitations associated with genetic manipulation and technically challenging approaches encouraged researchers to explore the use of small molecules to generate induced pluripotent stem cells. The first fully chemical derivation of iPSCs from murine fibroblasts was reported in 2013, through the employment of seven small-molecule compounds [189]. More recently, a completely chemical reprogramming of human cells has been described by Guan and colleagues, who applied this approach to both fetal and adult somatic cells [190], and subsequently refined it [191]. These new strategies represent significant progress, as they have made it possible to prevent the introduction into the cells of exogenous genetic materials, potentially reducing the risk of mutagenesis and improving the safety of future clinical applications. In the future, we envision that the synergy of chemical screening, genome editing techniques, single-cell RNAseq and computational tools will generate a better understanding of the molecular mechanisms that drive reprogramming [192]. From that knowledge, a new generation of efficient and safe protocols will be implemented [20].
